# Longitudinal evidence of the influence of early life circumstances, family characteristics, social ties and psychological distress on healthy behaviours of Brazilian adults: The Pro-Saude cohort study

**DOI:** 10.1371/journal.pone.0306565

**Published:** 2024-08-14

**Authors:** Mario V. Vettore, Tonje H. Stea, Rune Zahl-Olsen, Eduardo Faerstein

**Affiliations:** 1 Department of Dentistry and Oral Health, Aarhus University, Aarhus, Denmark; 2 Department of Health and Nursing Sciences, University of Agder, Kristiansand, Norway; 3 Department of Child and Adolescent Mental Health, Soerlandet Hospital, Kristiansand, Norway; 4 Department of Epidemiology, Institute of Social Medicine, State University of Rio de Janeiro, Rio de Janeiro, Brazil; Jimma University, ETHIOPIA

## Abstract

**Background:**

This study examined the influence of early life circumstances, family characteristics, social ties and psychological distress in adulthood on adult’s health-related behaviours.

**Methods:**

A cohort study (Pro-Saúde Study) involving technical and administrative civil servants at university campuses in Rio de Janeiro State, Brazil was conducted in Rio de Janeiro, Brazil. Data from 2155 adults were collected at baseline (1999) and after a 13-year period (2012–13). Family characteristics at 12 years of age were assessed retrospectively in 1999. Gender, marital status, living situation, social support, social networks of relatives and psychological distress were also measured in 1999. Data collection in 2012–13 included information about marital status, social networks of relatives, cigarette smoking, fruit and vegetable consumption and physical exercise. A conceptual model testing the relationships between variables was assessed through structural equation modelling.

**Results:**

Female gender (β = 0.043), better social networks of relatives in 1999 (β = 0.053) and 2012–13 (β = 0.069) and low psychological distress (β = -0.048) directly predicted less smoking. Better social networks of relatives in 2012–13 was directly linked to higher consumption of fruits (β = 0.045) and vegetables (β = 0.051) and being physically active (β = 0.070). Low psychological distress directly predicted higher fruit consumption (β = -0.040). Family characteristics at 12 years-old, marital status and living with other people were linked indirectly with health behaviours through social networks, social support and psychological distress.

**Conclusions:**

Adults with better early life family and social circumstances, and those who were married reported positive health behaviours through indirect pathways. Stronger social ties and lower psychological distress represented the pathways by which early life circumstances and relationship status influenced positive health behaviours.

## Introduction

Promoting healthy behaviours is among the core elements of public health policies to reduce the burden of non-communicable diseases (NCDs) which accounts for 41 million deaths annually, representing 71% of total deaths worldwide [1]. Tackling tobacco use, unhealthy diets, physical inactivity and harmful alcohol consumption has been identified as important components of cost-effective interventions to reduce the burden of NCD [2]. Conceptionally, health behaviours are recognized as key mediating mechanisms between socioeconomic factors and individual health outcomes [3].

There is sound evidence that environmental and individual factors are relevant determinants of health behaviours amongst adults [4]. Communities with lower density of bars and restaurants has been related to lower alcohol consumption, whereas increased access and availability of healthy food and beverages and investing in places for exercise and recreation, a safe infrastructure for active transport, and nature-based activities has been suggested to be strategies to address low levels of physical activity and obesity [5–7]. In addition, a diverse range of tobacco demand and control policies comprising tax and prices increases, advertising ban and smoke-free polices has effectively reduced smoking prevalence worldwide [8]. On the other hand, demographics, cultural beliefs and psychological factors are meaningful individual factors associated with health behaviours [9–11]. Physical activity barriers may include job strain, traditional roles for women related to family and domestic duties, and language barriers experienced by ethnic minority groups to participate in physical activity groups [9]. Age, differences in personal health concerns, low self-efficacy, lack of time and routine due to work demands can influence diet patterns, such as higher energy and fat intake, as well as smoking consumption [4]. Research also indicates that health-compromising behaviours are consistently more prevalent among individuals experiencing depression, anxiety and poor mental health [10, 11].

Socioeconomic factors, adverse childhood experiences, family composition, relationship status and social networks contribute to shape health-related behaviours across the life course [12–16]. During childhood and adulthood, social ties of relatives may promote positive behaviours through different mechanisms, including access to material resources and social support, and self-perceived health and psychological well-being [17–23]. Further, the intricated dynamics of the mechanisms preventing health risk behaviours are related to enhancing financial stability, developing and increasing social cohesion and social control, and strengthened sense of responsibility [21, 23].

Recent studies have substantially improved our understanding on how marital status and marital transitions may affect psychological well-being [20, 22] and health behaviours [12, 18, 22, 24] among adults. Their findings suggest that becoming and remaining married resulted in psychological benefits and positive health behaviours compared to those who were single or divorced [12, 18, 20, 22, 24]. Increased psychological distress and reduced social integration were also reported by those who became divorced/separated compared to individuals remaining married [20, 22]. Moreover, ending a relationship due to divorce or spouse’s death was associated with unhealthy behaviours [12, 18, 22, 24]. Research on the influence of early-life family composition indicated that parental divorce and not living with both parents was associated with long-term negative psychosocial effects [17, 19] and engagement in health risk behaviours [17, 25].

Previous studies examining the relationship of socioeconomic factors, family characteristics and relationship status with health behaviours in adulthood have predominantly used a cross-sectional design and did not assess early life and current family characteristics in the same study, preventing a comprehensive life course understanding of those relationships [12–14, 16]. The above-mentioned gaps in knowledge prompted us to carry out a robust longitudinal study to investigate the underlying mechanisms on the relationship between family composition and health behaviours. The aim of the present study was to test the influence of family and socioeconomic factors in childhood and marital status, living situation, social support, social networks and psychological distress in adulthood on adult’s health-related behaviours.

## Methods

### Sample

The study population included participants of the Pro-Saude Study, a prospective cohort study of socio-economic characteristics and health-related measures with technical and administrative civil servants at university campuses in Rio de Janeiro State, Brazil. Overall, the participants have sociodemographic characteristics similar to urban populations in developed countries [26]. Retired employees or those who were on non-medical leave were excluded.

Initially, all 4459 eligible employees were invited for the baseline data collection in between 01 August and 30 November 1999 (wave 1). Of these, 4030 participants were included (response rate = 90.4%). The second and third waves were conducted in 2001 (*n* = 3574, response rate = 80.2%) and 2007 (*n* = 3253, response rate = 73.02%), respectively. However, data from waves 2 and 3 were not applicable for the present study since the variables of interest were not evaluated in those waves. Wave 4 data collection occurred in 31March 2012 and 31 January 2014 when 3058 participants were re-evaluated (response rate = 68.6%), characterizing a 13-14-year interval period. We excluded all participants with incomplete data (*n* = 903), which resulted in a study population of 2155 adults (70.5% of wave 4) with complete data in 1999 and 2012–13. Further information about the Pro-Saude Study is available elsewhere [26].

### Theoretical model

**Fig 1:**
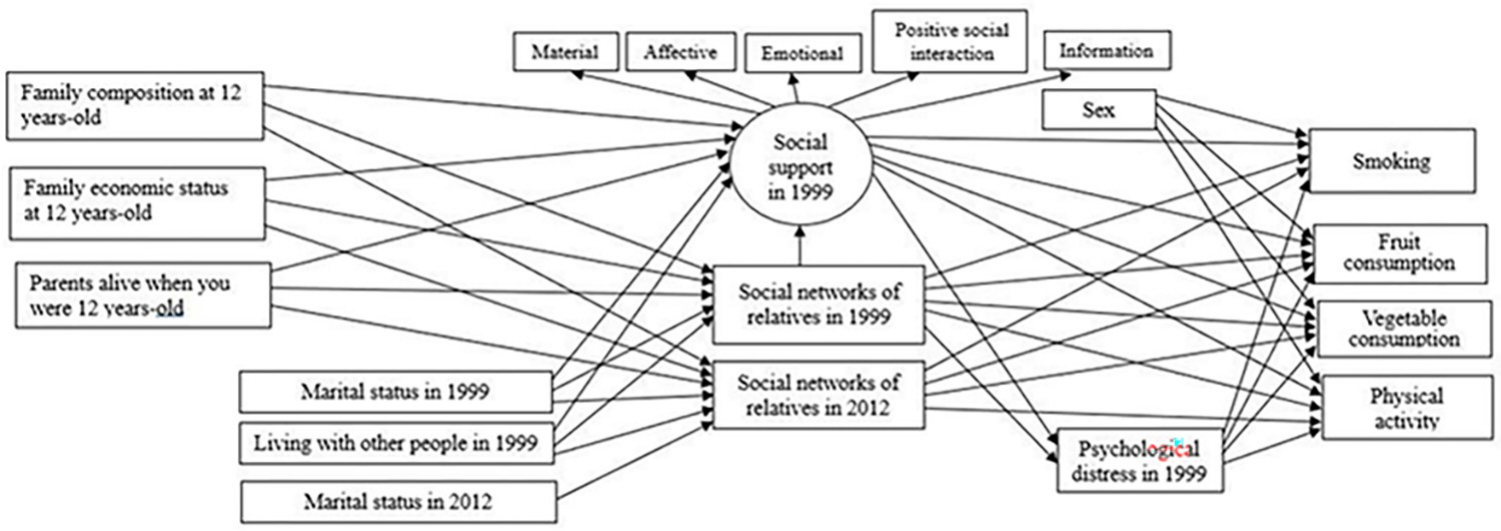
Full hypothesised model. Arrows represent hypothesised direct effects between variables.

A theoretical model involving the hypothesised relationships between adjacent and non-adjacent variables was adapted from a conceptual model for mechanisms linking social ties to health behaviours (Fig 1). According to Umberson’s conceptual model [12], structural social ties affecting health behaviours include social integration and social networks. In this study, structural social ties comprised of family members in childhood (e.g., parents) and adulthood (e.g., partners), and family socio-economic condition in childhood, indicating social integration, and social networks of relatives. In addition, health behaviours are influenced by content social ties including measures of social support and psychological distress [12]. It was hypothesised that structural social ties, including better family characteristics (e.g., living with both parents and having parents alive at 12 years-old) and higher family economic status in childhood, marital status related to more family attachments in adulthood (e.g., married) and living with other people would predict having greater social networks of relatives and greater social support. It was also expected that the social networks and social support measures would predict low psychological distress and healthier behaviours. In addition, we expected that female gender and lower psychological distress would directly predict better health-related behaviours. Thus, the mechanisms are suggested to operate through adjacent levels. Sex was also included in the model due to relevant sex-related disparities in health behaviours [16, 18, 24].

### Variables and measurement

The data were obtained through self-administered questionnaires filled out in the workplaces under supervision of trained field researchers. The selected items of the questionnaires are in the supplementary files (S1 Appendix).

Health-related behaviours were assessed in 2012–13 using self-reported measures of current cigarette smoking (1 = yes, 2 = no, stopped smoking < 1 year, 3 = no, stopped smoking ≥ 1 year, 4 = no, never smoked), frequency of fruit and vegetable consumption (1 = never or < 1 per week, 2 = 1–3 times per month, 3 = 1–3 times per week, 4 = 4–6 times per week, 5 = daily) and physical exercise to enhance participant’s health, physical condition or for the purpose of fitness or leisure in the last two weeks (1 = no, 2 = yes).

Family characteristics when the participant was 12 years old were assessed in 1999 and included family composition (1 = living with father and mother, 2 = living with mother or father only, 3 = living with other relatives, 4 = living in institution) and whether the participant’s parents were alive when he/she was aged 12 years (1 = father and mother alive, 2 = only father or mother was alive, 3 = both parents were not alive). Self-reported family economic status when the participant was 12 years old was assessed using a four-level ordinal scale (1 = rich, 2 = moderate, 3 = poor, 4 = very poor). This variable was based on participant’s perception of the family’s financial situation and the response options’ meanings were not explained to them. The variables sex (1 = male, 2 = female) was also measured in 1999. Family characteristics in adulthood were marital status in 1999 and 2012–13 (1 = married/living with partner, 2 = single, 3 = widow, 4 = separated/divorced). Marital status was an ordinal variable since divorced and widowed people are more predisposed to social isolation, whereas married people tend to have higher levels of social connectedness compared to other marital status groups [22]. Participants were also inquired whether they were living with other people in 1999 (1 = yes, 2 = no).

Social networks of relatives in 1999 and 2012–13 was measured following the methods used in the Whitehall study [27], by means of the number of family members the participants “feel comfortable with and who he/she can talk to about almost everything” [28]. Social support was assessed using the Brazilian validated version of the Medical Outcomes Study Social Support (MOS-SS) scale in 1999 [28, 29]. The MOS-SS scale comprises 19 items grouped into five dimensions of perceived availability of functional support: material, affective, emotional, positive social interaction and information. The higher score, the higher the social support. Social support was a latent variable represented by the scores of the five MOS-SS domains [28, 29]. Psychological distress was assessed in 1999 using the total score of the validated Brazilian version of the General Health Questionnaire-12 items (GHQ-12) [30, 31]. The respondents were asked about 12 recent symptoms and behaviours using a 4-point scale (1 = not at all), (2 = no more than usual), (3 = rather more than usual), (4 = much more than usual). The higher score, the worse the psychological distress.

### Test-retest reliability study

Internal consistency and reliability of the scales was assessed before baseline data collection with 1120 temporary civil servants not eligible to participate in the main study recruited at the same university campuses. Those civil servants were temporarily replacing others on leave not due to health reasons, and thus necessarily similar to the latter in sociodemographic aspects. The Cronbach coefficient of social support scale and GHQ-12 was 0.95 and 0.64. Kappa coefficients were 0.97 for cigarette smoking, 0.79 for frequency of fruit consumption, 0.67 for frequency of vegetables consumption and 0.63 for physical activity [32, 33]. Intra-class correlation coefficients for social support scale, social networks of family members and GHQ-12 were 0.88, 0.70 and 0.81 [28, 29]. The Cronbach coefficient of social support scale and GHQ-12 in the analysed sample was 0.96 and 0.75.

### Statistical analysis

The distribution of demographic characteristics, health-related behaviours, marital status, social support, social networks of relatives and psychological distress between participants with missing data (N = 903) and the studied sample (N = 2155) was compared using *t*-test (continuous variables) and Pearson Chi-square test (categorical variables).

Confirmatory factorial analysis (CFA) was used to assess the multidimensionality of social support latent variable and the correspondence with the proposed indicators in 1999. Standardized factor loadings, 95% confidence intervals (CIs) and standardized squared multiple correlations (*R*^2^) were estimated. Factor loadings > 0.30 were deemed acceptable. Structural equation modelling (SEM) examined the associations between variables according to the theoretical model. The maximum likelihood estimation method was used to estimate the standardised total, direct and indirect effects and respective 95% confidence intervals (CIs) between variables. The direct effect corresponds to a direct path from one variable to another, whereas the indirect effect encompasses a path mediated through other variables. Mediation was assessed according to the significance of the indirect effects. Non-significant paths were removed from the full model to generate a statistically parsimonious model. All analyses were conducted using STATA (StataCorp, version 17). The significance level established for all analyses was 5% (*p* < 0.05). The adequacy of the full and parsimonious models was evaluated through fit indices. Modification indices were used to improve the CFA and SEM model fit. A χ2/df < 3.0, standardized root mean square residual (SRMR) below 0.08, root mean square error of approximation (RMSEA) values below 0.06 and comparative fit index (CFI) and Tucker-Lewis index (TLI) above 0.90 were used to indicate an acceptable model fit [34].

### Ethics

The study was conducted according to the guidelines of the Declaration of Helsinki, and the research project was approved by the Research Ethics Committee of the Pedro Ernesto Teaching Hospital (Hospital Universitário Pedro Ernesto), Rio de Janeiro, Brazil. All participants provided informed written consent before data collection in all waves of the study.

## Results

Descriptive characteristics of the studied sample and participants excluded due to missing data are reported in Table 1. Respondents with complete data were older than those with missing data. The remaining variables did not differ between individuals with and without missing values. Participants’ average age in 1999 and 2012–13 was 40 years and 51.5 years, respectively. Women comprised 57.3% and 57.2% of the sample in 1999 and after 13–14 years follow-up. Frequency of non-smokers, daily fruit consumption, daily vegetable consumption and performed physical exercises during the previous two weeks in 1999 was 77.2%, 32.9%, 36.9% and 46.1%. In 2012–13, 87.1% were non-smokers, 38.6% and 22.1% had daily fruit and vegetable consumption and 42.7% performed physical exercises during the previous two weeks. Among the participants, 62% and 60% of the participants were married or living with a partner in 1999 and 2012–13, respectively (Table 2).

**Table 1:** Distribution of demographic characteristics, health-related behaviours, marital status, social support, social networks of relatives and psychological distress between excluded participants due to missing data and the studied sample.

Variable (year)	Excluded participants	Studied sample	*P*
*N*	903	2155	
Demographic characteristics (1999)			
Age (years), Mean (SD)	39.2 (8.4)	40.1 (8.2)	0.008
Sex, %			0.093
Male	46.2	42.7	
Female	53.8	57.3	
Health-related behaviours (2012)			
Current cigarette smoking, %			
Yes	12.5	12.9	0.786
No, stopped smoking < 1 year	1.8	1.9	
No, stopped smoking ≥ 1 year	19.6	25.3	
No, never smoked	66.1	59.9	
Fruits consumptions, %			0.588
Never or < 1 per week	5.1	3.7	
1–3 times per month	6.8	9.9	
1–3 times per week	25.4	31.8	
4–6 times per week	15.3	16.1	
Daily	47.5	38.5	
Vegetable consumption, %			0.216
Never or < 1 per week	4.9	2.2	
1–3 times per month	8.2	8.1	
1–3 times per week	19.7	30.0	
4–6 times per week	29.5	22.3	
Daily	37.7	37.4	
Physical exercise, %			
Yes	53.6	42.7	0.132
No	46.4	57.3	
Marital status (1999)			
Married or living with partner	60.6	61.9	0.248
Single	22.6	19.6	
Widow	2.8	2.8	
Divorced	14.0	15.7	
Marital status (2012)			0.188
Married or living with partner	60.0	60.3	
Single	7.1	2.8	
Widow	4.3	6.5	
Divorced	28.6	30.3	
Social support (1999), Mean (SD)	81.9 (16.9)	80.8 (17.9)	0.092
Social networks of relatives (1999), Mean (SD)	2.7 (2.0)	2.6 (2.0)	0.334
Social networks of relatives (2012), Mean (SD)	2.0 (1.7)	2.3 (1.8)	0.139
Psychological distress (1999), Mean (SD)	22.2 (5.4)	22.4 (5.5)	0.329

*p* refers to Pearson Chi-square test (categorical variables) and *t*-test (continuous variables)

* *p* < 0.05

**Table 2:** Demographic characteristics, marital status, living situation, social networks of relatives, and health-related behaviours at baseline (1999) and at 13-year follow-up (2012–13).

Variable	Baseline 1999	13-year follow-up 2012–2013
Age (years), Mean (SD)	40.1 (8.2)	51.5 (8.8)
Sex, %	42.7	42.3
Male	57.3	57.7
Female		
Marital status, %		
Married or living with partner	61.9	60.3
Single	19.6	2.8
Widow	2.8	6.5
Divorced	15.7	30.3
Living with other people, %		
Yes	90.3	88.8
No	9.7	11.2
Social networks of relatives, Mean (SD)	2.6 (2.0)	2.3 (1.8)
Health-related behaviours (2012)		
Current cigarette smoking, %		
Yes	22.8	12.9
No, stopped smoking < 1 year	1.3	1.9
No, stopped smoking ≥ 1 year	17.6	25.3
No, never smoked	58.3	59.9
Fruits consumptions, %		
Never or < 1 per week	6.1	3.7
1–3 times per month	14.3	9.9
1–3 times per week	33.1	31.8
4–6 times per week	13.7	16.1
Daily	32.9	38.5
Vegetable consumption, %		
Never or < 1 per week	2.8	2.2
1–3 times per month	9.6	8.1
1–3 times per week	30.2	30.0
4–6 times per week	20.4	22.3
Daily	36.9	37.4
Physical exercise, %		
Yes	46.1	42.7
No	53.9	57.3

**Fig 2:**
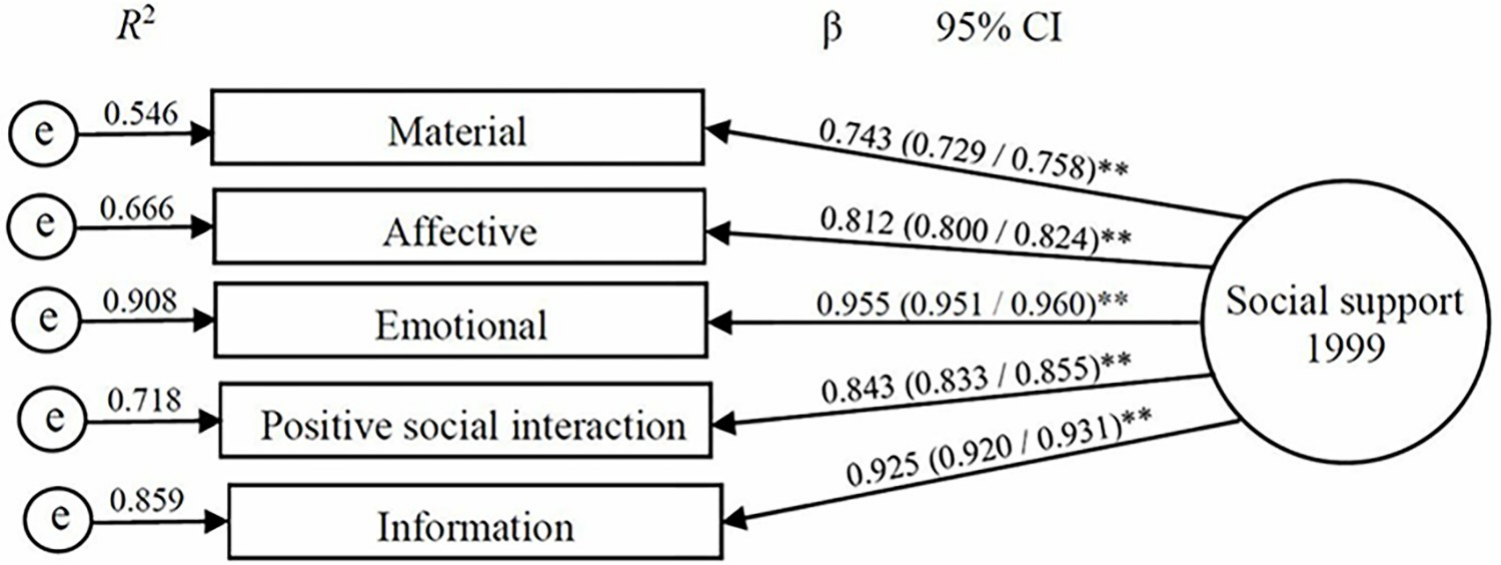
Confirmatory factor analysis of social support latent variable.

The standardized loading factors of social support latent variable obtained through CFA ranged from 0.743 (material support dimension) to 0.955 (emotional support dimension) and square multiple correlations ranged from 0.546 (material support) to 0.908 (emotional support) confirming the respective indicators (Fig 2).

Structural equation modelling supported the hypothesized model (full model). The parsimonious model was obtained after removing non-significant hypothesised paths of the full model. The full model and parsimonious model revealed adequate fit as the five of the a priori criteria were met (Table 3). The overall variance explained by the parsimonious model was 9.1%.

**Table 3:** Fit indices for the confirmatory factor analysis of full and parsimonious models.

Model	χ^2^ (d.f)	TLI	CFI	SRMR	RMSEA
Confirmatory factor analysis	1.931	0.997	0.996	0.004	0.038
Full	1.944	0.907	0.941	0.028	0.057
Parsimonious	1.812	0.908	0.924	0.033	0.057

**Fig 3:**
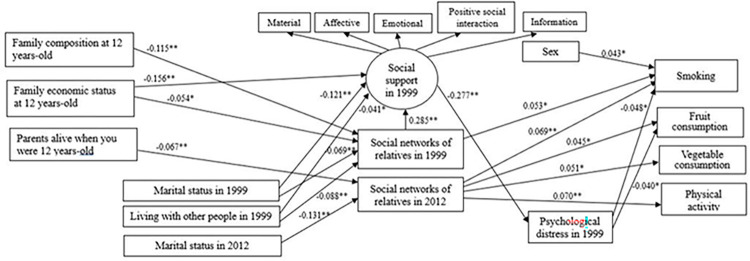
Parsimonious model depicting the direct effects between variables represented by solid lines. * *p* < 0.05, ** *p* < 0.001.

The direct effects of the parsimonious model were in the expected direction (Fig 3, S1 Table).

Being a non or former smoker was directly associated with female gender (β = 0.043), greater social networks of relatives in 1999 (β = 0.053) and 2012–13 (β = 0.069), and low psychological distress (β = -0.048). Higher frequency of fruit consumption was associated with greater social networks of relatives in 2012–13 (β = 0.045) and lower levels of psychological distress (β = -0.040). Greater social networks of relatives in 2012–13 was directly associated with higher frequency of vegetable consumption (β = 0.051) and being physically active (β = 0.070). Having greater social support was associated with lower psychological distress (β = -0.277). Greater social support was associated with lower family economic status at 12 years-old (β = -0.156), widowed and divorced marital status in 1999 (β = -0.121) and living alone (β = -0.041). Greater social networks of relatives in 1999 was associated with better family composition at 12 years-old (β = -0.115), better family economic status at 12 years-old (β = -0.054), marital status in 1999 (β = -0.069) and not living alone (β = -0.088). Not having one or both parents alive at 12 years-old (β = -0.067) and marital status in 2012–13 (β = -0.131) associated with less social networks of relatives in 2012–13 (Fig 3 and S1 Table).

**Fig 4:**
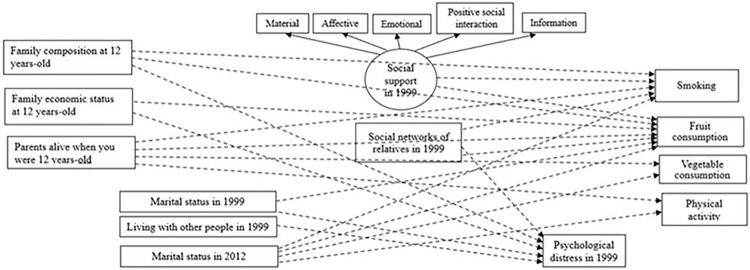
Significant indirect effects for the final statistically parsimonious model represented through dashed lines. * *p* < 0.05, ** *p* < 0.001.

Several significant indirect effects were observed (Fig 4 and S2 Appendix). Smoking was indirectly predicted by family composition at 12 years-old, parents alive at 12 years old, marital status in 2012–13 and social support. Frequency of fruit consumption was indirectly predicted by family economic status at 12 years-old, parents alive at 12 years old, marital status in 2012–13, social networks of relatives in 1999 and social support. Frequency of vegetable consumption was indirectly predicted by marital status in 2012–13. Being physically active was indirectly predicted by parents alive at 12 years old and marital status in 2012–13. Family composition at 12 years-old, family economic status at 12 years-old, marital status in 1999 and living with other people in 1999 were indirectly predicted by social support and psychological distress. Social networks of relatives in 1999 was indirectly predicted by psychological distress. S2 Appendix presents the different paths of the indirect pathways between non-adjacent variables in the parsimonious model.

## Discussion

The present findings support the relationships between early life and adult family characteristics, social ties, psychological distress and health-related behaviours among adults. Our results indicated that early life circumstances, including living with parents, better perception of family economic status and having parents alive at 12 years of age were associated with higher social support and greater social networks of relatives in adulthood. Social support and social networks of relatives were also meaningful characteristics related to marital status and living situation during adulthood. In addition, the greater social networks of relatives and the lower the psychological distress, the higher likelihood of being a non or former smoker, higher frequency of fruit and vegetable consumption and being physically active. More importantly, this study identified several important indirect pathways by which early life and adult family characteristics and social ties can influence health-related behaviours.

The effect of adverse childhood experiences on health-compromising behaviours during adulthood is undisputed [13, 15, 16]. Norwegian and US adults experiencing childhood difficulties, abuse and household dysfunction were more likely to being involved in health-risk behaviours, including unhealthy dietary habits, low level of physical activity and smoking cigarettes [13, 15, 16]. However, previous studies on that topic have evaluated childhood difficulties through a variety of adverse events during childhood or general questions using cross-sectional design, thus limiting direct comparisons across studies [13, 15, 16]. Our study validates previous findings and extends the literature by examining the social ties and psychological distress mechanisms by which specific unfavourable family characteristics during childhood influence health behaviours in adulthood.

On the other hand, there is evidence that being in a relationship is associated with healthy behaviours, as individuals who are married or planning to get married are less prone to smoke cigarettes and to report poor dietary habits [18, 22, 24]. One possible mechanism proposed to explain the positive influence of close relationships on health-related behaviours is the social protection hypothesis, which suggests that being in a relationship increase social support and social networks that are considered helpful resources in coping with stress and adopting and maintaining a healthy lifestyle [18, 22, 24]. The above-mentioned hypothesis is supported by previous longitudinal studies demonstrating that individuals who were continuously married and those who became married had lower risk of loneliness, depression, lower social integration and poor psychological well-being than those who unmarried and formerly married [20, 22]. To the best of authors’ knowledge, this is the first longitudinal study that has evaluated the social protection hypothesis addressing the pathways between marital status, social relationships, psychological distress and health-related behaviours amongst adults.

The traditional social foundations of health behaviours proposed by Durkheim support the contemporary evidence that a range of personal actions, such as poor diet, sedentarism and smoking explain a large proportion of deaths and NCDs [35]. In addition, the influence of social ties on health was consolidated as a social fact in the 1980’s [36] and a solid theoretical argument suggests that marriage has a symbolic meaning fostering a sense of purpose, commitment, and responsibility, motivating individuals to prioritize their health in order to better care for their loved ones [37]. Despite the remarkable increase in theory and research in this field, health behaviours are still acknowledged as a “black box” in the life course models on the influence of early life and current family characteristics, social ties and psychological well-being on health [12]. In this study, a theoretical model was developed according to the life course ‘accumulation of risk model’ that assumes that independent and clustered exposures that gradually accumulate over the life course have a great impact on health behaviours [38]. The proposed theoretical model was supported by the data and therefore should be considered a sound approach to investigate the role of early life socioeconomic factors, family composition and relationship status on adult’s health behaviours. The identified specific indirect paths revealed relevant sequences linking the predictors of health behaviours. Social networks of relatives, social support and psychological well-being were significant pathways, which indicates that health promotion strategies to reduce tobacco smoking, alcohol consumption, unhealthy dietary habits and physical inactivity should consider a psychological approach and involve family members and social ties rather than the individual approach.

### Strengths and limitations

This study benefits from the use of a theoretical model and structural equation modelling as analytical approach since existing research using longitudinal data have used traditional regression analyses and did not adopt theoretical models. Such aspects limit the application and interpretation of life course models since a theoretical model specifying the temporal order of exposure variables and their inter-relationships with specific health outcomes is essential in life course epidemiology [38]. The interpretation of our findings should consider several limitations. First, some predictors and health behaviours were measured only once throughout the study period. Therefore, such characteristics do not necessarily represent lifetime exposures or health behaviours trajectories. Second, the long duration of this cohort study and the use of self-administered questionnaires for data collection may explain the large number of excluded participants due to missing data and may have introduced some degree of selection bias. However, the characteristics included in the analysis did not differ significantly between the included and excluded participants. Third, participants may have not accurately remembered the investigated family characteristics at 12 years of age, which may have resulted in recall bias of family composition and family socioeconomic status at 12 years-old, and whether their parents were alive when they were 12 years-old. Fourth, our findings are based on a population of civil servants from Rio de Janeiro, Brazil, which may impose limitations to generalise our findings to adult populations with substantially different demographic and social characteristics.

According to our findings, which is also supported by results from previous studies, the following implications should be considered. Policies to tackle social inequalities during childhood may enhance social interaction during critical developmental periods facilitating the formation and maintenance of social relationships later in life [39]. Health promotion strategies should focus on unmarried people and those living alone with weak social ties due to lack of mutual support and encouragement to adopt healthy behaviours [12]. People with poor social ties of relatives should receive mental health support when presenting poor psychological well-being and the consequent discouragement in engaging in healthy behaviours [17].

## Conclusion

Our findings suggest that adult’s behaviour was directly influenced by social networks of relatives, social support and psychological status. Moreover, early life circumstances and marital status were distal determinants of health behaviours mediated by social networks of relatives, social support and psychological status. Thus, findings from the present study indicate that development of future health-related programs should focus on social determinants in preventing psychological distress and promoting healthy behaviours.

## Supporting information

S1 Appendix Selected items of the questionnaires. (DOCX)

S2 Appendix Indirect effects of the parsimonious model. (DOCX)

S1 Table Direct effects of the parsimonious model. (DOCX)
