# Supplementary material for: Longitudinal evidence of the influence of early life circumstances, family characteristics, social ties and psychological distress on healthy behaviours of Brazilian adults: The Pro-Saude cohort study
Source: PLoS One. 2024 Aug 14;19(8):e0306565. doi: 10.1371/journal.pone.0306565 (PMC11324140; doi:10.1371/journal.pone.0306565)
Supplement: S1 Appendix — (DOCX) [file pone.0306565.s001.docx]

**S1 Appendix. Selected items of the questionnaires**

**Demographics** (1999)

1. What is your sex?

( ) male ( ) female

**Health-related behaviours** (2012)

2. Do you currently smoke cigarettes?

( ) Yes

( ) No, stopped smoking < 1 year

( ) No, stopped smoking ≥ 1 year

( ) No, never smoked

3. How often do you eat fresh fruits?

( ) Never or < 1 per week

( ) 1-3 times per month

( ) 1-3 times per week

( ) 4-6 times per week

( ) Daily

4. How often do you eat vegetables?

( ) Never or < 1 per week

( ) 1-3 times per month

( ) 1-3 times per week

( ) 4-6 times per week

( ) Daily

5. In the last two weeks, have you engaged in any physical activity to improve your health, physical condition or for the purpose of fitness or leisure?

( ) no

( ) yes

**Family and socio-economic characteristics in childhood** (1999)

6. Who did you live when you were 12 years old?

( ) father and mother

( ) mother or father only

( ) other relatives

( ) institution

7. Were your parents alive when you were aged 12 years

( ) father and mother alive

( ) only father or mother was alive

( ) both parents were not alive

8. How was your family economic condition when you were 12 years old?

( ) rich

( ) moderate

( ) poor

( ) very poor

**Family characteristics in adulthood**

9. What is your current marital status? (1999)

( ) married or living with partner

( ) single

( ) widow

( ) divorced

10. What is your current marital status? (2012)

( ) married or living with partner

( ) single

( ) widow

( ) divorced

11. Are you living with other people (1999)

( ) yes

( ) no

**Social networks of family members**

12. How many family members do you feel comfortable with and can talk about almost everything? (1999) _______

13. How many family members do you feel comfortable with and can talk about almost everything? (2012) _______

**Medical Outcomes Study Social Support (MOS-SS) scale (1999)**

People sometimes look to others for companionship, assistance, or other types of support. How often is each of the following kinds of support available to you if you need it?

1. Someone to help you if you were confined to bed

( ) None of the time ( ) A little of the time ( ) Some of the time ( ) Most of the time ( ) All of the time

2. Someone you can count on to listen to you when you need to talk

( ) None of the time ( ) A little of the time ( ) Some of the time ( ) Most of the time ( ) All of the time

3. Someone to give you good advice about a crisis

( ) None of the time ( ) A little of the time ( ) Some of the time ( ) Most of the time ( ) All of the time

4. Someone to take you to the doctor if you needed it

( ) None of the time ( ) A little of the time ( ) Some of the time ( ) Most of the time ( ) All of the time

5. Someone who shows you love and affection

( ) None of the time ( ) A little of the time ( ) Some of the time ( ) Most of the time ( ) All of the time

6. Someone to have a good time with

( ) None of the time ( ) A little of the time ( ) Some of the time ( ) Most of the time ( ) All of the time

7. Someone to give you information to help you understand a situation

( ) None of the time ( ) A little of the time ( ) Some of the time ( ) Most of the time ( ) All of the time

8. Someone to confide in or talk to about yourself or your problems

( ) None of the time ( ) A little of the time ( ) Some of the time ( ) Most of the time ( ) All of the time

9. Someone who hugs you

( ) None of the time ( ) A little of the time ( ) Some of the time ( ) Most of the time ( ) All of the time

10. Someone to get together with for relaxation

( ) None of the time ( ) A little of the time ( ) Some of the time ( ) Most of the time ( ) All of the time

11. Someone to prepare your meals if you were unable to do it yourself

( ) None of the time ( ) A little of the time ( ) Some of the time ( ) Most of the time ( ) All of the time

12. Someone whose advice you really want

( ) None of the time ( ) A little of the time ( ) Some of the time ( ) Most of the time ( ) All of the time

13. Someone to do things with to help you get your mind off things

( ) None of the time ( ) A little of the time ( ) Some of the time ( ) Most of the time ( ) All of the time

14. Someone to help with daily chores if you were sick

( ) None of the time ( ) A little of the time ( ) Some of the time ( ) Most of the time ( ) All of the time

15. Someone to share your most private worries and fears with

( ) None of the time ( ) A little of the time ( ) Some of the time ( ) Most of the time ( ) All of the time

16. Someone to turn to for suggestions about how to deal with a personal problem

( ) None of the time ( ) A little of the time ( ) Some of the time ( ) Most of the time ( ) All of the time

17. Someone to do something enjoyable with

( ) None of the time ( ) A little of the time ( ) Some of the time ( ) Most of the time ( ) All of the time

18. Someone who understands your problems

( ) None of the time ( ) A little of the time ( ) Some of the time ( ) Most of the time ( ) All of the time

19. Someone to love and make you feel wanted

( ) None of the time ( ) A little of the time ( ) Some of the time ( ) Most of the time ( ) All of the time

**General Health Questionnaire- 12 items (1999)**

Now, we would like to know how you have been doing during the last two weeks concerning the following aspects. Here, we would like just to know about recent problems instead of those you might have had in the past.

“During the last two weeks, have you…”

1. Lost much sleep over worry?

( ) Not at all ( ) No more than usual ( ) Rather more than usual ( ) Much more than usual

2. Felt constantly under strain?

( ) Not at all ( ) No more than usual ( ) Rather more than usual ( ) Much more than usual

3. Been able to concentrate on what you’re doing?

( ) More than usual ( ) Same as usual ( ) Less than usual ( ) Much less than usual

4. Felt you were playing a useful part in things?

( ) More than usual ( ) Same as usual ( ) Less than usual ( ) Much less than usual

5. Been able to face up to your problems?

( ) More than usual ( ) Same as usual ( ) Less than usual ( ) Much less than usual

6. Felt capable of making decisions about things?

( ) More than usual ( ) Same as usual ( ) Less than usual ( ) Much less than usual

7. Felt you couldn’t overcome your difficulties?

( ) Not at all ( ) No more than usual ( ) Rather more than usual ( ) Much more than usual

8. Been feeling reasonably happy, all things considered?

( ) More than usual ( ) Same as usual ( ) Less than usual ( ) Much less than usual

9. Been able to enjoy your normal day-to-day activities?

( ) More than usual ( ) Same as usual ( ) Less than usual ( ) Much less than usual

10. Been feeling unhappy and depressed?

( ) Not at all ( ) No more than usual ( ) Rather more than usual ( ) Much more than usual

11. Been losing confidence in yourself?

( ) Not at all ( ) No more than usual ( ) Rather more than usual ( ) Much more than usual

12. Been thinking of yourself as a worthless person?

( ) Not at all ( ) No more than usual ( ) Rather more than usual ( ) Much more than usual
