# Supplementary material for: Longitudinal evidence of the influence of early life circumstances, family characteristics, social ties and psychological distress on healthy behaviours of Brazilian adults: The Pro-Saude cohort study
Source: PLoS One. 2024 Aug 14;19(8):e0306565. doi: 10.1371/journal.pone.0306565 (PMC11324140; doi:10.1371/journal.pone.0306565)
Supplement: S2 Appendix — (DOCX) [file pone.0306565.s002.docx]

**S3 Appendix. Indirect effects of the parsimonious model**

The total indirect effects are made up of specific indirect paths which were calculated as follows (all figures are standardized beta coefficients):

**Smoking**

1. **Family composition at 12 years-old to Smoking**

Family composition at 12 years-old → Social networks of relatives in 1999 → Smoking = -0.115 x 0.053 = -0.006

Family composition at 12 years-old → Social networks of relatives in 1999 → Social support → Psychological distress → Smoking = -0.115 x 0.285 x -0.277 x -0.048 = -0.0004

**2) Parents alive at 12 years-old to Smoking**

Parents alive at 12 years-old → Social networks of relatives in 2012 → Smoking = -0.067 x 0.069 = -0.005

**3) Marital status in 2012 to Smoking**

Marital status in 2012 → Social networks of relatives in 2012 → Smoking = -0.131 x 0.069 = -0.009

**4) Social networks of relatives in 1999 to Smoking**

Social networks of relatives in 1999 → Social support → Psychological distress → Smoking = 0.285 x -0.277 x -0.048 = 0.004

**5) Social support to Smoking**

Social support → Psychological distress → Smoking = -0.277 x -0.048 = 0.013

**Frequency of** **fruit consumption**

**6) Family economic status at 12 years-old to Fruit consumption**

Family economic status at 12-years old → Social support → Psychological distress → Fruit consumption = -0.156 x -0.277 x -0.040 = -0.002

Family economic status at 12 years-old → Social networks of relatives in 1999 → Social support → Psychological distress → Fruit consumption =-0.054 x 0.285 x -0.277 x -0.040 = -0.056

**7) Parents alive at 12 years old to Fruit consumption**

Parents alive at 12 years old → Social networks of relatives in 2012 → Fruit consumption = -0.067 x 0.045 = -0.003

**8) Marital status in 2012 to Fruit consumption**

Marital status in 2012 → Social networks of relatives 2012 → Fruit consumption 2012 = -0.131 x 0.045 = -0.006

**9)** **Social networks of relatives in 1999 to Fruit consumption**

Social networks of relatives in 1999 → Social support 1999 → Psychological distress 1999 → Fruit consumption = 0.285 x -0.277 x -0.040 = 0.003

**10) Social support to Fruit consumption**

Social support 1999 → Psychological distress → Fruit consumption = -0.277 x -0.040 = 0.011

**Frequency of vegetable consumption**

**11) Marital status in 2012 →** **Vegetable consumption**

Marital status in 2012 → Social networks of relatives in 2012 → Vegetable consumption = -0.131 x 0.051 = -0.007

**Physical activity**

**12) Parents alive when you were 12 years-old to Physical activity**

Parents alive when you were 12 years-old → Social networks of relatives in 2012 → Physical activity = -0.067 x 0.070 = -0.005

**13) Marital status in 2012 to Physical activity**

Marital status in 2012 → Social networks of relatives in 2012 → Physical activity = -0.131 x 0.070 = -0.009

**Social support**

**14) Family composition at 12 years-old to Social support**

Family composition at 12 years-old → Social networks of relatives in 1999 → Social support = -0.115 x 0.285 = -0.033

**15) Family economic status at 12 years-old to Social support**

Family economic status at 12 years-old → Social networks of relatives in 1999 → Social support = -0.054 x 0.285 = -0.015

**16) Living with other people to Social support**

Living with other people → Social networks of relatives in 1999 → Social support = -0.088 x 0.285 = -0.025

**17) Marital status in 1999 to Social support**

Marital status in 1999 → Social networks of relatives in 1999 → Social support = -0.069 x 0.285 = -0.020

**Psychological distress**

**18) Family composition at 12 years-old to Psychological distress**

Family composition at 12 years-old → Social networks of relatives in 1999 → Social support → Psychological distress = -0.115 x 0.285 x -0.277 = 0.009

**19) Family economic status at 12 years-old to Psychological distress**

Family economic status at 12 years-old → Social support → Psychological distress = -0.156 x -0.277 = 0.043

Family economic status at 12 years-old → Social networks of relatives in 1999 → Social support → Psychological distress = -0.054 x 0.285 x -0.277 = 0.004

**20) Social networks of relatives in 1999 to Psychological distress**

Social networks of relatives in 1999 → Social support → Psychological distress = 0.285 x -0.277 = -0.079

**21) Marital status 1999 to Psychological distress**

Marital status 1999 → Social support → Psychological distress = -0.121 x -0.277 = 0.034

Marital status 1999 → Social networks of relatives in 1999 → Social support → Psychological distress = -0.069 x 0.285 x -0.277 = 0.006

**22) Living with other people to Psychological distress**

Living with other people → Social support → Psychological distress = -0.041 x -0.277 = 0.011

Living with other people → Social networks of relatives in 1999 → Social support → Psychological distress = -0.088 x 0.285 x -0.277 = 0.007
