# Supplementary material for: Longitudinal evidence of the influence of early life circumstances, family characteristics, social ties and psychological distress on healthy behaviours of Brazilian adults: The Pro-Saude cohort study
Source: PLoS One. 2024 Aug 14;19(8):e0306565. doi: 10.1371/journal.pone.0306565 (PMC11324140; doi:10.1371/journal.pone.0306565)
Supplement: S1 Table — (DOCX) [file pone.0306565.s003.docx]

**S2 Table. Direct effects of the parsimonious model**

|  | **β** | **95% CI** | **P** |
| --- | --- | --- | --- |
| **Smoking** |  |  |  |
| Sex → Smoking | 0.043 | 0.001 / 0.085 | 0.044 |
| Social networks of relatives in 1999 → Smoking | 0.053 | 0.011 / 0.095 | 0.014 |
| Social networks of relatives in 2012 → Smoking | 0.069 | 0.027 / 0.111 | 0.001 |
| Psychological distress → Smoking | -0.048 | -0.090 / -0.006 | 0.026 |
| **Fruit consumption** |  |  |  |
| Psychological distress → Fruit consumption | -0.040 | -0.076 / -0.003 | 0.033 |
| Social networks of relatives in 2012 → Fruit consumption | 0.045 | 0.003 / 0.087 | 0.036 |
| **Vegetable consumption** |  |  |  |
| Social networks of relatives in 2012 → Vegetable consumption | 0.051 | 0.010 / 0.094 | 0.017 |
| **Outcome: Physical activity** |  |  |  |
| Social networks of relatives in 2012 → Physical activity | 0.070 | 0.028 / 0.112 | 0.001 |
| **Outcome: Psychological distress** |  |  |  |
| Social support → Psychological distress | -0.277 | -0.317 / -0.237 | < 0.001 |
| **Social support** |  |  |  |
| Family economic status at 12 years-old → Social support | -0.156 | -0.195 / -0.116 | < 0.001 |
| Marital status in 1999 → Social support | -0.121 | -0.162 / -0.080 | < 0.001 |
| Living with other people → Social support | -0.041 | -0.082 / -0.001 | 0.049 |
| Social networks of relatives in 1999 → Social support | 0.285 | 0.258 / 0.324 | < 0.001 |
| **Social networks of relatives** in **1999** |  |  |  |
| Family composition at 12 years-old → Social networks of relatives in 1999 | -0.115 | -0.157 / -0.073 | < 0.001 |
| Family economic status at 12 years-old → Social networks of relatives in 1999 | -0.054 | -0.097 / -0.012 | 0.013 |
| Marital status in 1999 → Social networks of relatives in 1999 | -0.069 | -0.111 / -0.027 | 0.001 |
| Living with other people in 1999 → Social networks of relatives in 1999 | -0.088 | -0.130 / -0.046 | < 0.001 |
| **Social networks of relatives in 2012** |  |  |  |
| Marital status in 2012 → Social networks of relatives in 2012 | -0.131 | -0.172 / -0.090 | < 0.001 |
| Parents alive at 12 years-old → Social networks of relatives in 2012 | -0.067 | -0.109 / -0.026 | 0.002 |
